# Incidence of Asymptomatic Gallstones in Obese Patients Who Underwent Bariatric Surgery in Qassim Region at King Fahad Specialist Hospital

**DOI:** 10.7759/cureus.44154

**Published:** 2023-08-26

**Authors:** Bandar Saad Assakran, Renad Khalid, Hala Albadrani, Aishah Alsuhaibani, Asrar Almutairi, Raghad Alhomidan, Ghayda Alfarhan, Ream Alshaya

**Affiliations:** 1 General Surgery, King Fahad Specialist Hospital, Buraydah, SAU; 2 College of Medicine, Qassim University, Buraydah, SAU; 3 Department of Medicine, Unaizah College of Medicine and Medical Sciences, Unaizah, SAU

**Keywords:** laparoscopic cholecystectomy, asymptomatic gallstones, bariatric surgery, obesity, gallstones

## Abstract

Background: Gallstone is a significant health issue in the KSA and other developing countries. Multiple important risk factors have been identified as being associated with gallstones. Obesity is one of the risk factors for gallstone formation. Therefore, this study intends to determine the incidence of asymptomatic gallstone disease among the obese population in the Qassim region. The purpose of this study is to determine the incidence of asymptomatic gallstone in obese patients and the risk factors that contribute to its development in the Qassim region. As well as to compare the prevalence of gallstone disease between age groups and genders.

Methodology: A retrospective study of all patients who underwent bariatric surgery and had gallstones between January 2018 and January 2022 at King Fahad Specialist Hospital in Qassim Region, Saudi Arabia. The data, including age, gender, body mass index (BMI), and co-morbidities, will be collected from their charts.

Results: The current study included 295 patients with a mean age of 34.83 years (SD = 11.7) and 126 (42.7%) male participants. The most common comorbidity was diabetes mellitus, which was present in 54 (18.4%) participants, followed by hypertension in 42 (14.3%) participants. Of the 295 participants, 232 (78.6%) had asymptomatic gallstones, while 63 (14.3%) patients were symptomatic. The results showed that younger people (16-25 years) had the highest odds ratio of having asymptomatic gallstones compared to the reference group (>55 years). Gender was also significantly associated with asymptomatic gallstones, with males having higher odds of having asymptomatic gallstones than females. Participants with comorbidities other than diabetes mellitus had lower odds of having asymptomatic gallstones.

Conclusion: The present study's main finding is that obese patients receiving bariatric surgery had a significant prevalence of comorbidities and asymptomatic gallstones. According to the results, diabetes mellitus, male gender, and younger age may all be risk factors for the occurrence of asymptomatic gallstones in this population.

## Introduction

Cholelithiasis refers to the presence of abnormal concretions (gallstones) in the gallbladder. Gallstones are common and greatly contribute to health care costs and patient morbidity, yet death directly due to complications of gallstones is rare today [1]. The prevalence of gallstones reaches 10%-20% of the adult population in developed countries, with peak incidence among those who are older than 40 years old. There are many theories about the causes of gallstones, such as imbalance in bile salts, lecithin, cholesterol, calcium carbonate, and bilirubin, biliary stasis is a key component in gallstone formation and impaired gallbladder emptying. Many risk factors can contribute to the formation of gallstones, which includes obesity, insulin resistance, dyslipidemia, female sex multiparity or pregnancy, age (> 40 years of age) and family history [2]. Gallstones are the most common risk factor for admission to an emergency room worldwide; they are known to affect 60%-70% of American Indians [2]. Among white adults in Western countries, it has an incidence of 10% [3]. However, it is frequently a disease that is recognized at a late stage, as it remains asymptomatic in nearly 80% of cases [4]. For the last few years, many studies about the prevalence and risk factors of gallstones have been carried out around the world. A study that included 1001 participants (2015) in Iraq recorded a prevalence of 13.5% of asymptomatic gallstones [5]. In KSA (2017), a study that recruited 500 subjects revealed a prevalence of 8.6% among the general Saudi population. Many risk factors were associated with a higher prevalence of gallstone disease, including physical inactivity and obesity. The study revealed that 67.4% of gallstone patients were obese [6]. However, scant data are present in the Qassim region regarding the incidence of asymptomatic gallstones in obese patients. Therefore, this study intends to ascertain the incidence of asymptomatic gallstone disease among the obese population in the Qassim region.

## Materials and methods

This hospital-based retrospective descriptive study aims to determine the incidence of asymptomatic gallstones in obese patients who underwent bariatric surgery at King Fahad Specialist Hospital in Buraydah city, Qassim Region, Saudi Arabia, between January 2018 and January 2022. The study was conducted for four years, and a sample size of more than 384 subjects is planned to be collected. The inclusion criteria for the study are patients who underwent bariatric surgery and had gallstones, and the exclusion criteria include patients with coeliac disease, chronic kidney disease, chronic liver disease, and those who previously underwent bariatric surgery.

Data for the study was collected from the electronic medical records of eligible patients. A standardized data collection form was used to collect variables related to the study objectives, such as age, sex, BMI, type of bariatric surgery, laboratory and radiological findings, and treatment for gallstones. The collected data was entered into a password-protected database on a secure server, and patient identifiers were removed to maintain confidentiality. Descriptive statistics was used to analyze the collected data, and the association between variables was determined using chi-square tests with a level of statistical significance set at p < 0.05.

A pilot study was conducted on a small sample of 10-15 patients to ensure the feasibility of the study, refine the data collection form, and identify any challenges related to data collection. The study used a convenience sampling technique, and eligible patients will be identified by searching the EMRs of patients who underwent bariatric surgery and had gallstones at KFSH. This study was conducted in accordance with the ethical principles outlined in the Declaration of Helsinki. The study protocol was submitted to the Qassim Region Research Ethics Committee for approval, and patient confidentiality will be maintained throughout the study. The data collected was used for research purposes only.

## Results

In the current study, we were able to collect the data for 295 patients who had a history of undergoing bariatric surgery and had gallstones between January 2018 and January 2022 at King Fahad Specialist Hospital. Table 1 presents the demographic factors of the participants in the study. The mean age of the participants was 34.83 years (SD = 11.7), with the majority falling in the age range of 26-35 years (32.5%). The age range with the least number of participants was >55 years (5.1%). In terms of gender, there were 126 (42.7%) male participants and 169 (57.3%) female participants. The mean BMI of the participants was 44.89 (SD = 7.08), indicating that the participants were severely obese.

**Table 1:** Demographic factors of the participants

		Count	Column N %	
Age	Mean (SD)	34.83 (11.7)		
Age	18-25	76	25.8%	
	26-35	96	32.5%	
	36-45	64	21.7%	
	46-55	44	14.9%	
	> 55	15	5.1%	
Gender	Male	126	42.7%	
	Female	169	57.3%	
BMI	Mean (SD)	44.89 (7.08)		
	Overweight	1		0.34%
	Class I obesity	12		4.07%
	Class II obesity	64		21.69%
	Class III obesity	218		73.90%

**Figure 1:**
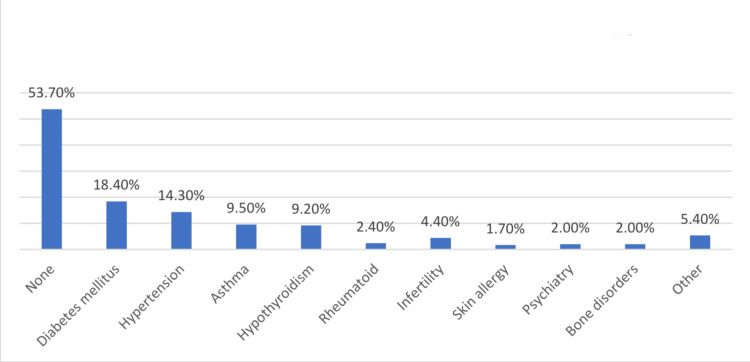
The prevalence of comorbidities among the participants

Figure 1 shows the prevalence of comorbidities among the participants in the study. Of the 295 participants, 136 (46.3%) had at least one comorbidity. The most common comorbidity was diabetes mellitus, which was present in 54 (18.4%) participants, followed by hypertension in 42 (14.3%) participants. Asthma was present in 28 (9.5%) participants, and hypothyroidism was present in 27 (9.2%) participants. Other less common comorbidities reported in the study included rheumatoid (2.4%), infertility (4.4%), skin allergy (1.7%), psychiatry (2.0%), and bone disorders (2.0%). There were also 16 (5.4%) participants who reported having other comorbidities. Of the 295 participants, 232 (78.6%) had asymptomatic gallstones, while 63 (21.4%) were symptomatic.

In Table 2, the results of the study revealed that younger age (16-25 years) was associated with the highest odds ratio of having asymptomatic gallstones compared to the reference group (>55 years). Specifically, individuals in the 16-25 age group had an odds ratio of 4.96 (95% CI: 1.42-17.2) for having asymptomatic gallstones. Gender was also found to be significantly associated with asymptomatic gallstones, with males having higher odds of having asymptomatic gallstones than females. Specifically, males had an odds ratio of 2.65 (95% CI: 1.42-4.94) for having asymptomatic gallstones compared to females.

**Table 2:** Association between having asymptomatic gallstones and demographic factors and having different comorbidities

		Symptomatic or not				Odd ration of having asymptomatic gall stones			P-value
		Symptomatic		Asymptomatic					
		Count	Row N %	Count	Row N %	OR		95 % CI	
Age	16-25	9	11.8%	67	88.2%	4.96		1.42-17.2	0.003*
	26-35	14	14.6%	82	85.4%	3.90		1.20-12.6	
	36-45	20	31.3%	44	68.8%	1.46		0.45-4.68	
	46-55	14	31.8%	30	68.2%	1.43		0.42-4.80	
	> 55	6	40.0%	9	60.0%	Reference			
Gender	Male	16	12.7%	110	87.3%	2.65		1.42-4.94	0.002*
	Female	47	27.8%	122	72.2%	Reference			
Having other comorbidities	Yes	38	27.7%	99	72.3%	0.48		0.27-0.86	0.013*
	No	25	15.8%	133	84.2%	Reference			
Diabetes mellitus	No	46	19.1%	195	80.9%	1.97		1.00-3.76	0.045*
	Yes	17	31.5%	37	68.5%	Reference			
Hypertension	No	52	20.6%	201	79.4%	1.37		0.64-2.91	0.409
	Yes	11	26.2%	31	73.8%	Reference			
Asthma	No	57	21.3%	210	78.7%	1.00		0.38-259	0.992
	Yes	6	21.4%	22	78.6%	Reference			
Hypothyroidism	No	59	22.0%	209	78.0%	0.61		0.20-185	0.384
	Yes	4	14.8%	23	85.2%	Reference			
Rheumatoid	No	60	20.8%	228	79.2%	2.85		0.62-13.07	0.106
	Yes	3	42.9%	4	57.1%	Reference			
Infertility	No	60	21.3%	222	78.7%	1.1		0.29-4.12	0.877
	Yes	3	23.1%	10	76.9%	Reference			
Skin allergy	No	63	21.7%	227	78.3%	0.32		0.02-5.96	0.240
	Yes	0	0.0%	5	100.0%	Reference			
Psychiatry	No	60	20.8%	229	79.2%	3.82		0.75-19.4	0.084
	Yes	3	50.0%	3	50.0%	Reference			
Bone disorders	No	63	21.8%	226	78.2%	0.27		0.02-4.93	0.197
	Yes	0	0.0%	6	100.0%	Reference			
Other	No	57	20.4%	222	79.6%	2.33	0.82-6.69		0.105
	Yes	6	37.5%	10	62.5%	Reference			

Participants with comorbidities other than diabetes mellitus had lower odds of having asymptomatic gallstones. In fact, having other comorbidities was associated with a lower odds ratio of 0.48 (95% CI: 0.27-0.86) for having asymptomatic gallstones. On the other hand, diabetes mellitus was found to be significantly associated with higher odds of having asymptomatic gallstones. Participants with diabetes mellitus had an odds ratio of 1.97 (95% CI: 1.00-3.76) for having asymptomatic gallstones compared to those without diabetes. However, the study did not find significant associations between asymptomatic gallstones and hypertension, asthma, hypothyroidism, rheumatoid arthritis, infertility, skin allergies, psychiatry, bone disorders, and other comorbidities.

## Discussion

The purpose of the current study was to find out how common comorbidities and asymptomatic gallstones were in obese patients who had bariatric surgery. The study discovered that the sample population was significantly affected by comorbid conditions, with diabetes mellitus and hypertension being the most prevalent. The study also found that most patients had asymptomatic gallstones, with a higher likelihood of asymptomatic gallstones being connected with younger age and male gender.

Obese people frequently have comorbid conditions; hence, bariatric surgery is frequently suggested as a treatment to help them live healthier lives [7,8]. In the current study, 46.3% of patients had at least one comorbid condition, which is consistent with earlier studies that found a high prevalence of comorbidities among obese people receiving bariatric surgery [9-11]. The current study's high prevalence of diabetes mellitus and hypertension is also in line with earlier studies [12-14], emphasizing the significance of managing these comorbidities in the management of obesity.

With 78.6% of the patients having asymptomatic gallstones, the prevalence of asymptomatic gallstones in the current study was also high. This result is in line with earlier studies that showed a significant prevalence of asymptomatic gallstones in obese people [15-17]. The incidence of asymptomatic gallstones in the current study shows that bariatric surgery patients may need to undergo postoperative surveillance for gallstones [18]. Bariatric surgery is known to increase the risk of acquiring gallstones [19,20]. However, it is still unknown how to treat asymptomatic gallstones in this population, and more study is required to establish the best course of action.

The current study also found several participant characteristics linked to asymptomatic gallstones. The likelihood of having asymptomatic gallstones was observed to increase with younger age and male gender. The fact that having asymptomatic gallstones increases with age is in line with earlier studies [21-24]. Although the exact cause of why being younger increases the incidence of asymptomatic gallstones is unknown, it has been postulated that hormonal changes during puberty may be involved [25]. Additionally, the result that men are more likely to have asymptomatic gallstones is in line with earlier studies [26]. The causes of this gender disparity are unclear; however, it has been hypothesized that estrogen may increase the pain of gall stones, increasing the prevalence of symptomatic gall stones among females [27].

The current study also discovered that having diabetes mellitus increased the likelihood of having symptomatic gallstones. This result is in line with earlier studies that indicated a higher risk of symptomatic gallstones in those with diabetes mellitus [28]. In addition, it was found that the current study discovered that participants' probabilities of having asymptomatic gallstones were lower among those who had comorbidities other than diabetes mellitus. This discovery is a little expected, and it could be because comorbid conditions may increase the sensitivity of the patients to pain, making them more at risk of developing symptomatic gallstones [29]. To fully comprehend the connection between these illnesses and the incidence of asymptomatic gallstones, more investigation is necessary.

When interpreting the findings, it is important to consider the limitations of the current study. The study's sample size was relatively limited, and it was carried out at just one institution. Furthermore, there was no control group of non-obese participants in the study, which makes it difficult to make generalizations about the population. Finally, the study did not evaluate the clinical outcomes of these patients; it merely investigated the occurrence of asymptomatic gallstones.

## Conclusions

The present study's main finding is that obese patients receiving bariatric surgery had a significant prevalence of comorbidities and asymptomatic gallstones. According to the results, diabetes mellitus, male gender, and younger age may all be risk factors for the occurrence of asymptomatic gallstones in this population. The findings of this study could have an impact on how bariatric surgery patients are managed, as well as how people who are more likely to develop asymptomatic gallstones are identified. The current study also discovered that having diabetes mellitus increased the likelihood of having symptomatic gallstones, and this may influence the guidelines for doing cholecystectomy with bariatric procedures in diabetic patients. Further research is required to determine the best management of asymptomatic gallstones in this population and to further understand the mechanisms behind these relationships.
